# Comparing Endovascular Approaches in Lower Extremity Artery Disease: Insights from a Network Meta-Analysis

**DOI:** 10.3390/jcm13041024

**Published:** 2024-02-10

**Authors:** Reka Aliz Lukacs, Lisa Ingrid Weisshaar, Daniel Tornyos, Andras Komocsi

**Affiliations:** 1 Department of Interventional Cardiology, Heart Institute, Medical School, University of Pécs, 7624 Pécs, Hungary; lukacs.reka@pte.hu (R.A.L.); tornyos.daniel@pte.hu (D.T.); 2 Klinikum Leverkusen Medical Clinic 1, 51375 Leverkusen, Germany; lisaweisshaar@yahoo.de

**Keywords:** peripheral artery disease, chronic limb ischemia, infrapopliteal, femoropopliteal, endovascular intervention, network meta-analysis

## Abstract

Background: Endovascular therapy offers an alternative for treating femoropopliteal (FP) and infrapopliteal (IP) lesions related to occlusive lower extremity artery disease. Despite numerous trials, the effectiveness of restenosis prevention using local drug delivery devices remains a topic of debate. Objectives: An updated systematic review and network meta-analysis was conducted. Our overall aim was to summarize the most recent clinical evidence regarding endovascular approaches for FP and IP atherosclerotic lesions. Methods: We conducted a search for randomized trials in the MEDLINE database, and extracted data related to clinical endpoints. Our primary focus was on the rate of major adverse events (MAEs), including mortality, amputation, and target lesion revascularization (TLR). A multiple treatment network meta-analysis supplemented with component network analyses was performed to examine the impact of combined treatment. Results: Our search yielded 33 randomized controlled trials encompassing 5766 patients. This included 19 studies focused on femoropopliteal and 14 on IP lesions, accounting for 3565 and 2201 patients, respectively. Drug-coated balloons (DCBs) and drug-eluting stents (DESs) displayed a reduced MAE risk in comparison to plain old balloon angioplasty (POBA)—RR for DCB: 0.64 (95% CI: 0.52–0.77) and for DES: 0.71 (95% CI: 0.51–0.99). The bare-metal stent (BMS) group manifested the most substantial MAE risk, being 59% higher relative to the DCB cohort (BMS vs. DCB RR: 1.59; 95% CI: 1.03–2.47). For FP lesions, DES was the standout performer, curtailing MAE risk by 55% relative to POBA. Within IP lesions, DES mitigated the MAE risk by 25% versus POBA. DCB did not exhibit any notable MAE reduction when pitted against POBA. Conclusion: In FP arteries, both DESs and DCBs yielded significantly diminished MAEs, thus outpacing other techniques. Regarding IP arteries, only DESs resulted in significantly fewer MAEs. In alignment with contemporary research, our findings revealed no signs of elevated mortality in patients undergoing treatment with drug-eluting apparatuses.

## 1. Introduction

Peripheral arterial disease (PAD) is a prevalent and debilitating disorder affecting millions worldwide. The World Health Organization states that over 200 million people globally are afflicted by PAD, with the majority residing in North America and Europe. In the United States, PAD’s prevalence is approximately 8–12% among individuals 65 years and older, reflecting a growing patient population [1]. PAD is typified by the emergence of obstructive arterial lesions within the lower extremities, which limit vascular perfusion and, in severe cases, can culminate in total vascular occlusion.

Endovascular therapy (EVT) presents as a viable alternative to surgical reconstruction for managing occlusive lower extremity artery disease, especially for femoropopliteal (FP) and infrapopliteal (IP) lesions. However, the efficacy of balloon angioplasty, with or without concurrent stent implantation, is substantially undermined by the occurrence of local restenosis.

With the variability in patients’ anatomical and physiological characteristics, there is a growing recognition of the importance of individualized therapeutic choices. Tailoring treatment decisions based on specific patient attributes, including their anatomical peculiarities, not only enhances the therapeutic outcomes but may also minimize potential complications. The realm of PAD treatment is gradually shifting from a one-size-fits-all approach to a more nuanced, personalized strategy. This individualization becomes especially pertinent when selecting patients for EVT versus surgery and further discerning the optimal EVT modality, whether it is the use of balloon dilation, stents, drug-eluting stents (DESs) and drug-coated balloons (DCBs). The decision matrix is intricate, considering factors such as the need for stent scaffolding or the application of local drug delivery for restenosis prevention.

In coronary interventions, DESs and DCBs have shown significant promise in preventing restenosis. Yet, for peripheral arteries, the advantages of these drug delivery strategies are less apparent, highlighting a distinction in their efficacy between coronary and peripheral arterial lesions [2]. Despite numerous clinical trials, the efficacy of DES and DCB in preventing restenosis in FP or IP lesions continues to be debated. There is a burgeoning interest in DES and DCB as potential treatments for PAD, but comparative data on their effectiveness and safety remain scant.

In this study, we undertook a network meta-analysis (NMA), collating the latest clinical evidence on endovascular interventions. Our objective was to offer an exhaustive review of the evidence, facilitating clinical decision making for PAD patients. We incorporated long-term data to assess sustained efficacy. Trials focusing on FP and IP atherosclerotic lesions were analyzed in subgroups to delve deeper into local anatomical differences. Additionally, we employed a multiple treatment NMA and a component NMA (CNMA) to discern the effects of stent implantation from those induced by the local delivery of an antiproliferative drug.

## 2. Materials and Methods

We conducted a comprehensive review of literature to identify relevant clinical trials examining the safety and efficacy of drug-eluting devices, specifically DESs or DCBs in patients with FP or IP lesions. Systematic searches spanned PubMed, Embase, and Cochrane Library. We considered articles published from the inception of these databases up to 1 May 2023. Keywords encompassed “Peripheral Artery Disease”, “Chronic limb ischemia”, “infrapopliteal”, “femoropopliteal”, “Endovascular”, “PTA”, “BMS”, “DCB”, “DEB”, “Everolimus”, “Paclitaxel”, and “Sirolimus”.

To ensure thorough coverage, reference lists of identified studies were screened for potential inclusions. Two authors independently evaluated the search results based on established inclusion and exclusion criteria (RL and LIW), with a third author (AK) consulted for disagreements.

Inclusion criteria consisted of (a) availability of full-text articles; (b) randomized controlled trial design; (c) comparative analysis between at least two treatment options: PTA, BMS, DCB, and/or DES, applied to (d) FP or IP arteries, and clearly defined intervention site inclusion criteria; (e) evaluation of safety or efficacy of endovascular treatment for peripheral artery disease or critical limb ischemia; and (f) a minimum observation period of six months. Studies were excluded if they were non-randomized, did not report results based on anatomical localization, were single-arm studies, failed to provide comprehensive data, compared endovascular approaches with non-endovascular ones, or were duplicate publications. The quality of included trials was appraised using the Cochrane risk-of-bias tool.

We adhered to the PICOS framework for our literature search and data extraction: the patient population (P) were patients diagnosed with peripheral arterial disease who underwent an EVT; the intervention (I) was the use of “BMS”, “DCB”, and/or “DES”; the comparison (C) was against PTA; the outcome (O) considered was the combined effect on major adverse events (MAEs), which include mortality, amputation, and target lesion revascularization (TLR).

The data extracted prioritized intention-to-treat analysis results with the longest follow-up times. If numerical data were not directly provided, but graphical data were, we estimated values using a digital screen ruler.

The MAEs were variably defined across studies, using internal definitions as per individual trial protocols. To address potential heterogeneity, we checked these definitions against our predefined definition of clinical failure, which is a composite of mortality, limb loss, and persisting or re-emerging objective symptoms in the limb. Secondary outcomes encompassed individual MAE components, restenosis rates, and major and minor amputations.

We utilized multiple-treatment NMA for the four different treatment variations (PTA, BMS, DCB, DES). Data analysis incorporated frequentist methods for an NMA using the R environment and the *netmeta* package [3]. Treatment effects were estimated by synthesizing direct and indirect evidence, and treatments were ranked using the surface under the cumulative ranking curve. Risk ratios (RR) and standard errors were calculated from individual study data and incorporated into the NMA model. Trial heterogeneity and inconsistency were explored using the Cochrane Q test and I^2^ statistic.

Given the dual-action nature of DES—involving potential risks and benefits related to both stent implantation and local antiproliferative treatment—it was important to evaluate the influence of each component. Thus, we implemented an additive model assuming that the effect of combined treatments equaled the sum of the effects of its individual components. Conforming to these, we pre-specified the use of multiple-treatment NMA supplemented with component NMA modeling, utilizing an additive model function [4]. Subgroup analyses excluding either FP or IP studies were conducted using the same statistical methods. All tests were two-tailed, with *p*-values < 0.05 denoting statistical significance.

The protocol for this review was registered with the PROSPERO (International Prospective Register of Systematic Reviews) database. Data evaluation was performed in accordance with the PRISMA extension statement for the reporting of systematic reviews, which incorporate NMA of healthcare interventions [5].

## 3. Results

**Figure 1:**
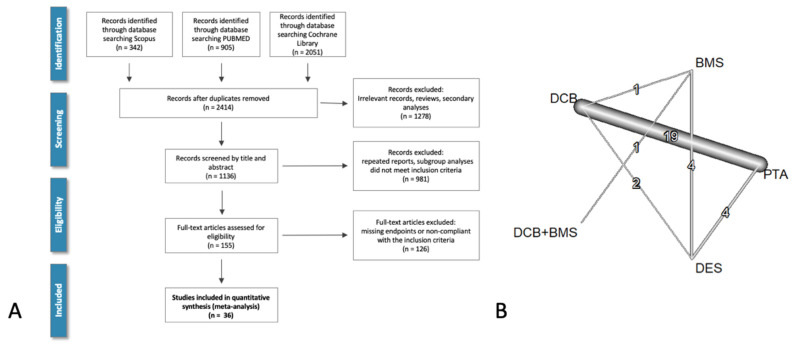
Literature search and evidence network. Panel (**A**). PRISMA flowchart of the literature search. Panel (**B**). Net-Graph for MAEs (major adverse events) in the full model analysis. In this diagram, the nodes represent the treatment arms, while the edges show direct comparisons (with thickness representing the number of the studies included).

Our systematic review identified 33 studies, incorporating a total of 5745 patients that fulfilled the predefined inclusion criteria (Figure 1, Table S1). These included five studies comparing DES vs. PTA, five contrasting DES vs. BMS, and two studies evaluating DES vs. DCB. Furthermore, 19 studies compared DCB vs. PTA, one study compared DCB vs. BMS, and another study compared DCB + BMS vs. BMS.

All enrolled patients were diagnosed with PAD. The majority of patients were elderly (>65 years old) and presented with risk factors such as diabetes, hypertension, or hypercholesterolemia. The most common follow-up period across these studies was twelve months. Follow-up periods varied, with seven studies at six months, three at twenty-four months, one at nine months, three at three years, and two at five years. Patient characteristics are summarized in Supplementary Material Table S2. Net heat plots and net-split analyses suggested no major inconsistencies with respect to consistency. Further, low publication bias was observed, as demonstrated by funnel plot analysis (Figures S3 and S4).

### 3.1. Clinical Outcomes

#### 3.1.1. MAE

Major adverse events (MAEs) were reported in 31 studies, with a total of 1371 occurrences. Of these, 662 cases were identified in IP studies and 709 in FP studies. The MAEs were defined variably across studies, using internal definitions as per individual trial protocols.

**Figure 2:**
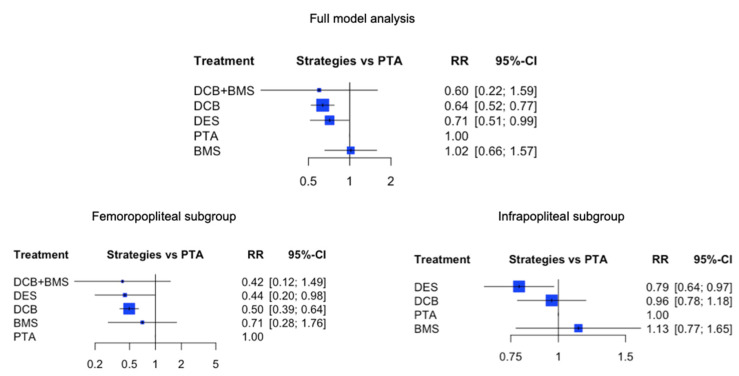
Results of the risk of MAE in full mode analysis and in femoropopliteal and infrapopliteal subgroups using different endovascular treatments. The forest plots depict the results of the network meta-analysis computed based on direct and indirect comparisons of risk ratio (RR) and 95% confidence intervals (95% CI). Data are presented as comparisons of percutaneous transluminal angioplasty (marked as ‘PTA’). Abbreviations: MAEs (major adverse events); BMS (bare-metal stent); DES (drug-eluting stent); DCB (drug-coated balloon); RR (risk ratio); CI (confidence interval).

In the comprehensive model analysis, DCB demonstrated the most favorable outcome, reducing the risk of MAEs by 36% compared with PTA. This difference was statistically significant (RR: 0.64; 95% confidence interval (CI) [0.52; 0.77], *p* < 0.001). DES also led to a reduced risk of MAE compared with PTA (RR: 0.71; 95% CI [0.51; 0.99], *p* = 0.048). Compared with DCB, the risk associated with BMS was 59% higher (BMS vs. DCB RR: 1.59; 95% CI [1.03; 2.47], *p* = 0.036). However, data consistency was moderate I^2^ 58.5% [37.0%; 72.7%], with no significant heterogeneity observed either within or between designs (*p* values < 0.0001). DES exhibited the best treatment outcomes in both FP and IP lesion subgroups, while the BMS treatment had the highest MAE risk (Figure 2).

#### 3.1.2. Mortality

Out of the 31 studies, 504 deaths were reported. Among them, 384 deaths were recorded in IP studies, and 120 in FP studies. According to the comprehensive model analysis, BMS had the lowest mortality risk, but this did not reach statistical significance (RR 0.78, 95% CI [0.54; 1.13], *p* = 0.19). Mortality risk differences in other treatment groups were within 10%, and none were statistically significant (DCB vs. PTA: RR 0.96, 95% CI [0.77; 1.19]; DES vs. PTA: RR 0.95, 95% CI [0.80; 1.12]). The data were consistent and homogeneous (I2 0%; *p* = 0.94).

In the FP lesions subgroup, BMS had the lowest mortality risk, which was statistically significant (RR 0.21, 95% CI [0.05; 0.96], *p* = 0.04). In contrast, the IP subgroup did not have any statistically significant findings or results that deviated significantly from those of the comprehensive model analysis (Figure S2).

#### 3.1.3. Major Amputation, TLR, Restenosis Rate, and Late Lumen Loss

In the comprehensive model analysis, the risks of major amputation and TLR did not differ by more than 10%, rendering these findings not statistically significant and without apparent clinical relevance. In the IP subgroup analysis, the DES group had the lowest amputations risk, though this was not statistically significant. BMS had the highest risks of major amputation and TLR, nearly doubling the risk compared to PTA. When compared to PTA, the risk of major amputation with DCB was similar. Furthermore, the risk of TLR in the DES group was comparable to that of the PTA group.

Although the DCB exhibited a 30% lower risk of TLR compared to the PTA and DES treatments, this was not statistically significant (Table 1).

#### 3.1.4. Treatment Ranking Analysis

In the comprehensive analysis focused on the MAE endpoint, DCB emerges as the most effective, reflected by its leading P-score of 0.80, contrasting starkly with BMS’s P-score of 0.16 at the lower end of the spectrum. Delving into the FP subgroup for MAE, DES takes precedence with a P-score of 0.76, marginally superseded by DCB + BMS at 0.74. Meanwhile, PTA trails with a P-score of 0.09. Venturing into the IP subgroup, the MAE metric shows joint top-tier placement of BMS and DES, both achieving a P-score of 0.96.

DES maintains a recurrent upper-tier ranking, notably in the realms of MAE and major amputation endpoints. Specifically, within the comprehensive model targeting amputations, DES dominates with a P-score of 0.80. BMS, exhibiting some fluctuations across various endpoints, persistently portrays superior performance within the mortality domain; the full model showcases its P-score to be 0.83, peaking further in the FP subgroup at 0.91. On the other side of the spectrum, PTA predominantly occupies lower ranks, with a discernible trend in the MAE metric across models.

**Figure 3:**
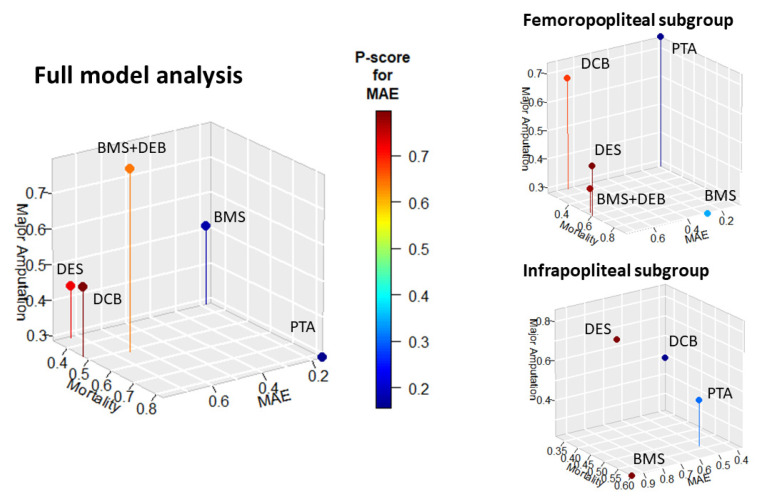
Results of treatment ranking. Three-dimensional efficacy visualization of treatments across endpoints and subgroups. Each plot delineates the treatment landscape across three primary endpoints, mortality (*x*-axis), major amputation (*y*-axis), and major adverse events (MAEs, *z*-axis), for the full model, femoropopliteal (FP), and infrapopliteal (IP) subgroups. Dots are positioned in the 3D space based on their performance across these endpoints, with their color intensity reflecting the P-score of MAEs. Each subgroup is presented in a separate graphic for clarity.

Treatments integrating local drug delivery mechanisms (DES, DCB, and DCB + BMS) consistently ascend the rankings, with a marked prominence in MAE and amputation metrics. Analyzing the FP subgroup concerning MAE, both DES and DCB + BMS are laudably efficacious, registering P-scores of 0.76 and 0.74, correspondingly. Similarly, the IP subgroup’s evaluation places both DES and BMS at the pinnacle, each recording a P-score of 0.96 (Figure 3, Table S3).

#### 3.1.5. Component Analysis

Upon evaluating the influence of various components on MAEs, it was discerned that local drug delivery significantly attenuated risk across all models. In the comprehensive model, a risk diminution of 32% was ascertained. In the FP and IP subgroups, the observed risk abatements were 38% and 30%, respectively.

In terms of mortality outcomes, none of the components, be they local drug delivery, angioplasty, or stent scaffolding, demonstrated a statistically significant impact on any of the examined models.

**Figure 4:**
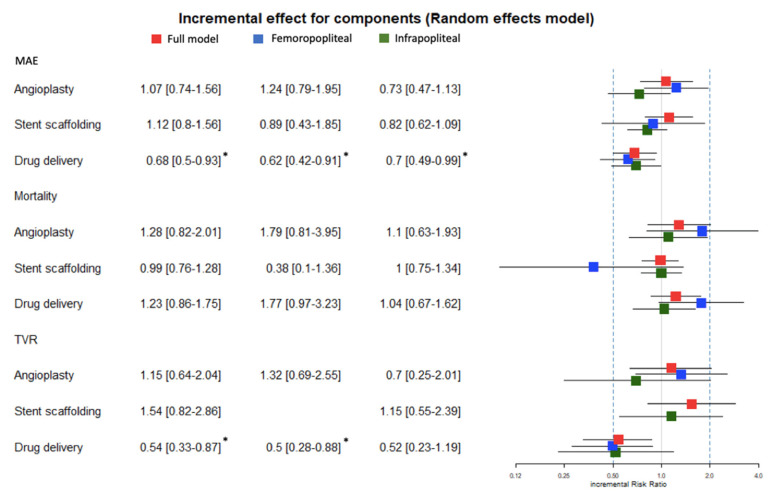
Results of Component Analysis. The figure presents a forest plot detailing the incremental risk ratio (iRR) values attributed to angioplasty, stent scaffolding, and local drug delivery across three distinct analyses: major adverse events (MAEs), mortality, and target vessel revascularization (TVR). The results from the comprehensive analysis, alongside the femoropopliteal and infrapopliteal subgroups, are each displayed in separate columns. Within the forest plot, iRR values are represented with corresponding 95% confidence intervals. Results are stratified by analysis type, with data from the comprehensive analysis represented by red boxes, the femoropopliteal subgroup shown in blue boxes, and the infrapopliteal subgroup in green boxes. * indicates that they are significant.

Regarding TVR, the efficacy of local drug delivery was again underscored. In the overarching model, a significant risk reduction of 46% was identified and attributed to drug delivery. This pattern persisted in the FP model, wherein a substantial 50% reduction was observed. Although the IF model suggested a 48% risk decrement associated with drug delivery, this did not meet the level of statistical significance. Importantly, the incremental benefits purported by the angioplasty and stent scaffolding components failed to achieve statistical significance in all the analytical models (Figure 4).

## 4. Discussion

The introduction of endovascular treatment for occlusive atherosclerosis in the lower limbs has significantly revolutionized the management of peripheral artery disease (PAD). Historically, PAD has primarily been managed through open surgical procedures, often accompanied by significant morbidity and extended recovery times.

The local anatomy plays a vital role in determining the most suitable intervention for each patient. For longer calcified lesions, in cases wherein a conduit vein is available, bypass grafting is a fitting option; meanwhile, in cases of shorter lesions and in patients at higher surgical risk, EVT is the preferred approach. The optimal treatment strategy should be determined on a case-by-case basis, taking into account the specific clinical and anatomical features of the individual patient [6,7].

Recent advances in technology, technique, and pharmaceuticals have pushed endovascular interventions to the forefront as a less invasive and equally effective option for treating PAD. However, it should be noted that based on the BEST-CLI trial, in advanced stages among patients with CLTI who had an adequate great saphenous vein for surgical revascularization, the incidence of a major adverse limb event or death was significantly lower in the surgical group than in the endovascular group [8]. Endovascular procedures involve the use of catheters to access and treat arterial lesions, offering considerable benefits including reduced patient discomfort, shortened hospital stays, and lower complication rates compared to traditional surgical approaches. This shift in treatment methods emphasizes a crucial evolution in the understanding and management of lower extremity occlusive atherosclerosis.

Consequently, a thorough understanding of the various endovascular therapies and their comparative effectiveness is crucial for optimizing PAD management. Local drug delivery devices, such as DCBs and DESs, have been designed to address the challenges associated with restenosis following endovascular intervention. Several studies have already dealt with the cost–benefit ratio in patients hospitalized for PAD who underwent revasculariztion [9], comparing endovascular intervention with bypass surgery [10], as well as different types of stents and balloons among endovascular methods [11]. However, among patients with symptomatic lower extremity PAD undergoing endovascular revascularization, initial treatment costs and total 2-year costs vary significantly according to clinical and lesion-level characteristics, as well as symptom burden [12].

### Local Drug Delivery

In recent years, numerous studies comparing different EVT modalities have been published. In these trials, DES showed predominantly promising results, while the benefits of treatments with DCBs remain hotly debated. Data on long-term outcomes were available for a fragment of these trials, placing procedural success and short-term efficacy at the forefront of these discussions. Our aim was to combine results from randomized controlled trials (RCTs) with different treatment options in one analysis by conducting an NMA. This allowed us to include direct and indirect comparisons to analyze several treatment options simultaneously in a mixed-treatment comparison network [13,14].

In the comprehensive analysis, both DCB and DES demonstrated favorable outcomes in comparison to other treatments, particularly regarding MAEs. DCB treatment exhibited the lowest risk of MAEs, reducing this risk by 35% compared to PTA. This finding was statistically significant, highlighting the effectiveness of DCB in managing PAD. Similarly, DES treatment also showed beneficial results by reducing the risk of MAEs compared to PTA. This lends further support to the notion that local drug delivery devices can be more effective than other treatments in managing PAD, potentially due to their ability to locally inhibit cellular processes leading to restenosis, thus prolonging the efficacy of the intervention. However, potential risks including the development of ISR, thrombosis, and other stent-related complications, coupled with increased costs compared to traditional stents, warrant careful consideration. Routine monitoring and follow-up are integral for maintaining the stent’s efficacy and promptly addressing any complications.

These results underline the potential advantages of using local drug delivery devices in endovascular interventions for PAD. However, further investigation and long-term studies may be required to better understand the full extent of their benefits and potential limitations. Earlier NMAs in the field of EVT aimed to consolidate the results of these trials. However, some of them concentrated only on short-term but not long-term data over a period of 3 years [15,16,17]. Earlier studies in line with our results also reported the advantages of DCB and DES regarding revascularization [18]. Based on these data, recommendations of primary stenting in TASC type D lesions and the use of DCB, especially in complex and calcified lesions, have been proposed [19,20]. While our findings are consistent with the general trends observed in earlier meta-analyses, our analysis did not indicate the superiority of any specific treatment in terms of total amputation and TLR. This discrepancy that may be attributed to the inclusion of long-term results in our study.

It is also important to note that one study, published by Kuno et al. in 2020, discovered an excess mortality rate among patients treated with DES. This publication opened an ongoing discussion about mortality in patients with paclitaxel-eluting devices [17]. In terms of mortality, in line with the latest analyses, no statistically significant difference was observed among the different treatment groups in the full model analysis. However, in the FP subgroup, BMS demonstrated the lowest mortality risk reaching a level of statistical significance.

According to the most recent 2017 ESC Guidelines on PAD, EVT is recommended as the initial treatment of choice for shorter lesions [6]. Anatomical location in addition to the lesions’ characteristics may substantially influence the long-term efficacy of EVT. In the FP segment, the arterial walls are subjected to external forces such as compression, torsion, and bending, which can pose a significant challenge to successful treatment. IP arteries, due to their small size and predisposition to calcification, also pose unique challenges, making them more difficult to access and treat. Additionally, IP lesions may be multiple and elongated, further complicating the EVT procedure [21,22].

In FP arteries, DES and DCB had the best outcomes, with a significantly lower number of MAEs. The use of DCB preparation before BMS implantation (DCB + BMS) has also shown promising results, especially regarding TLR. Other recent studies have revealed a similar trend. Zhou et al. also found an advantage of DCB for TLR, supporting that FP lesions should be treated with stent technologies and directional atherectomy plus DCB [23].

EVTs have promising results, as multiple trials and NMAs have shown, but there remain problems with in-stent restenosis (ISR) and long segment occlusions in IP arteries [15,16,24,25,26,27,28]. Stenting, especially in IP arteries, needs to be carefully accessed. Stents should be avoided in areas suitable for potential bypasses. Moreover, ISR is more complicated to treat than restenosis after plain balloon angioplasty [7]. In the 2014 IN.PACT.DEEP trial, Zeller et al. found an increased risk of major amputation within the first year post procedure when comparing DCB to PTA in patients with CLI due to IP lesions [29]. In the follow-up study published in 2020, they reported no superiority regarding safety and effectiveness and no significantly higher risk of amputation or mortality in the DCB group [30]. Katsanos et al. conducted a meta-analysis in 2020 that investigated the risk of death and amputation when using paclitaxel DCB in IP arteries. The analysis found a higher risk of death upon amputation in the first year after treatment [31].

Contrarily, a meta-analysis by Ipema et al. revealed no significant increase in mortality or amputation rate when comparing DCB to PTA control in patients with IP lesions [24]. In addition, a recent study from 2021 comparing paclitaxel-coated devices with non-coated devices found no increase in mortality in the group with eight paclitaxel-coated devices in FP arteries after a follow-up time of 60 months [32].

Regarding the risk of major amputation and TLR, no significant differences were noted among the treatment strategies in the full model analysis. Nevertheless, the DES group had the lowest risk of major amputation in the IP subgroup, albeit without reaching statistical significance.

Furthermore, in the subgroup analysis of studies treating IP lesions, DES treatment was associated with the lowest risk of major amputation. Although this result did not reach statistical significance, it suggests a potential advantage of DES in managing more severe diseases or more distal lesions, which often present greater treatment challenges.

These data underline the need for more data regarding the long-term efficacy of EVT modalities.

## 5. Limitations

Several limitations of our study merit discussion. Our analysis was confined to studies published in English. Consequently, potential ethnic and national disparities might introduce bias into our results. Furthermore, our selection criteria restricted the analysis to randomized controlled trials. This selection process could potentially amplify the risk of publication bias.

Moreover, the majority of the studies had a follow-up duration ranging from 6 to 12 months. A lengthier follow-up period of three to five years was reported by only three studies, leaving uncertainty around the evidence regarding the prognosis over one year.

Given that an NMA juxtaposes direct and indirect data, it may be more prone to bias compared to traditional meta-analyses. While study characteristics were well matched across studies, a lack of blinding and considerable variations in lesion length should be noted.

Owing to pronounced heterogeneity and missing data, we refrained from analyzing data pertaining to restenosis rate and late lumen loss. Additionally, inconsistencies were observed in reports of the duration of antiplatelet therapy and the specific medications used post intervention, which did not allow for analyses of the influence of these potential confounders. The clinical relevance and the positive aspects of our research lie in the large-scale meta-analysis of RCTs, revealing the advantages and disadvantages of different EVTs concerning various endpoints. This provides an opportunity to facilitate clinical decision making for PAD patients.

## 6. Conclusions

Our network meta-analysis (NMA) examined 14 infrainguinal (IP) and 19 femoropopliteal (FP) randomized controlled trials (RCTs), incorporating long-term data. The comprehensive analysis demonstrated that local drug delivery devices conferred a 40–29% reduction in relative risk (RR) of adverse events compared to balloon angioplasty. Specifically, within the femoropopliteal arteries, drug-eluting stents (DESs) and drug-coated balloons (DCBs) exhibited the most favorable outcomes, demonstrating a significant reduction in adverse events. Notably, this reduction was more pronounced with the DCB + BMS combination. However, due to the limited number of cases, analyses involving this option yielded wide confidence intervals and did not achieve statistical significance. Consequently, additional data are required to accurately assess the benefits and potential risks associated with this treatment modality. For IP arteries, DES emerged as the most efficacious treatment, marked by a substantial reduction in MAE events, albeit without prior results for TLR. Even though mortality and major amputation rates reflected a decrease, the reductions were not statistically significant. Echoing recent findings, this NMA did not identify a rise in mortality among patients treated with drug-eluting devices.

In summary, DES therapy offers potential for considerable improvements in symptom management and disease progression in PAD patients. DES not only minimizes the risk of amputation but also ameliorates patients’ quality of life. DES also outperforms traditional BMS in terms of restenosis rates.

DCBs represent a less invasive alternative to DESs, as they offer local drug delivery without the necessity of implanting a stent. Like DESs, they aim to prevent ISR by delivering the drug directly into the arterial wall. DCBs present certain advantages over DESs and a reduced necessity for long-term antiplatelet treatment. Nevertheless, DCBs might not exhibit the same efficacy as DESs, and their long-term outcome data are somewhat limited.

When considering DCBs or DESs for PAD treatment, a careful assessment of potential risks and benefits is crucial. Further studies are warranted to fully comprehend the comparative effectiveness of these treatment options in PAD patients.

## Figures and Tables

**Table 1:** Results of the endpoints in full model analysis. The table depicts the results of the network meta-analysis computed based on direct and indirect comparisons of risk ratio (RR) and 95% confidence intervals (95% CI). Abbreviations: BMS; bare-metal stent, DCB; drug-coated balloon; DES; drug-eluting stent, MAEs; major adverse events, TLR; target lesion revascularization. * indicates that they are significant.

Comparison	BMS	DCB	DCB + BMS	DES
MAE	1.02 (0.66; 1.57)	0.64 (0.52; 0.77) *	0.60 (0.22; 1.59)	0.71 (0.51; 0.99) *
Mortality	0.78 (0.54; 1.13)	0.96 (0.77; 1.19)	1.50 (0.14; 16.49)	0.95 (0.80; 1.12)
Major amputation	1.50 (0.47; 4.74)	1.04 (0.64; 1.69)	1.50 (0.03; 87.61)	0.78 (0.50; 1.21)
Minor amputation	1.02 (0.20; 5.27)	1.07 (0.80; 1.44)		0.99 (0.60; 1.61)
Restenosis	1.25 (0.63; 2.48)	0.50 (0.37; 0.67) *	0.42 (0.10; 1.70)	0.60 (0.36; 1.00)
TLR	1.33 (0.65; 2.71)	0.47 (0.35; 0.62) *	0.68 (0.16; 2.82)	0.72 (0.39; 1.33)

## Data Availability

No new data were created or analyzed in this study.

## Supplementary Materials

The following supporting information can be downloaded at: https://www.mdpi.com/article/10.3390/jcm13041024/s1, Table S1: Study characteristics. Table S2: Patient characteristics. Figure S1: Results of the network analysis of mortality endpoints in full model analysis and subgroups. Figure S2: Results of the network analysis of mortality endpoints in full model analysis and subgroups. Figure S3: Net Split Plot Analysis of Major Adverse Events in Full Model Analysis. Figure S4: Comparison Adjusted Funnel Plot for Major Adverse Event Endpoint in Full Model Analysis. Figure S5: Risk of bias in studies.
