# Supplementary material for: Comparing Endovascular Approaches in Lower Extremity Artery Disease: Insights from a Network Meta-Analysis
Source: J Clin Med. 2024 Feb 10;13(4):1024. doi: 10.3390/jcm13041024 (PMC10889479; doi:10.3390/jcm13041024)

Supplementary material

to the article entitled **"Comparative Assessment of Drug Eluting Stents and Balloons in the Treatment of Peripheral Arterial Disease: Risks, Benefits, and Future Directions – a network meta-analysis of randomized trials"**

Tartalomjegyzék

Supplementary material *1*

Table S1 Study characteristics **2**

Table S2 Patient characteristics **6**

Figure S1 Results of the network analysis of mortality endpoints in full model analysis and subgroups **9**

Figure S2: Net Heat Plot Analysis of Treatment Efficacy in Peripheral Arterial Disease 10

Figure S3: Net Split Plot Analysis of Major Adverse Events in Full Model Analysis **11**

Figure S4: Comparison Adjusted Funnel Plot for Major Adverse Event Endpoint in Full Model Analysis **12**

Figure S5: Risk of bias in studies **13**

Table S1 Study characteristics

Abbreviations: BMS (bare meal stent); DCB (drug coated balloon); DES (drug eluting stent); PTA (percutan transluminal angioplasty); FP (femoropopliteal); IP (infrapopliteal); PAD (peripheral artery disease); CLI (critical limb ischeamia); R (Rutherfort); DM (diabetes mellitus); ASS =(Aspirin); C (Clopidogrel); NA (not available)

**Author, year, acronym**	**Follow up**	**Comparison**	**No. Sample Size**	**Subgroup**	**Target Group**	**postprocedure antithrombitc therapie**
**Bausback 2017** *RANGER-SFA*	6 months	DCB-PTA	105	FP	PAD (R2-4)	Dual 1 month; one antiplatelet drug daily
**Bausback 2019** Real PTX	3 years	DES-DCB	150	FP	PAD (R2-5)	Dual < 2 months; ASS daily
**Dake 2011** *Zilver PTX*	12 months	DES-BMS	479	FP	PAD (R>1)	Dual 60 days; ASS daily
**Duda 2006** *SIROCCO*	24 months	DES-BMS	93	FP	PAD (R1-4)	Dual 1 month; ASS at least 12 month
**Falkowski A. 2020**	3 years	DES-BMS	256	FP	PAD (R2-5)	Dual 2 month; ASS daily
**Iida 2018** *MDT-2113 SFA*	12 months	DCB-PTA	100	FP	PAD (R2-4)	Dual 1 month (without stent) 3 months (bailout with stent); ASS >6month
**Jia 2016** *AcoArt-I*	12 months	DCB-PTA	200	FP	PAD (R2-5)	Dual 6 month
**Krisnan 2017** *ILLUMENATE Pivotal*	12 months	DCB-PTA	300	FP	PAD (R2-4)	Dual 1 month; ASS dialy for 12 month
**Liistro 2013** *DEBATE-SFA*	12 months	DCB+BMS-BMS	104	FP	PAD (Claudication or CLI)	Dual 1 month (BMS) 3 months

						(DCB+BMS); ASS: daily
**Rosenfield 2015** *LEVANT-2*	12 months	DCB-PTA	476	FP	PAD (R2-4)	Dual 1 month; ASS daily
**Scheinert 2014** *LEVANT-1*	24 months	DCB-PTA	101	FP	PAD (R2-5)	Dual 1 month (without stent) 3 months (bailout with stent); ASS daily
**Scheinert 2015** *BIOLUXP-1*	12 months	DCB-PTA	49	FP	PAD (R2-5)	Dual 1 month (without stent) 3 months (with stent)
**Schroeder 2017** *ILLUMENATE EU RCT*	12 months	DCB-PTA	294	FP	PAD (R2-4)	Dual 1 month (without stent) 3 months (bailout with stent); ASS daily
**Steiner 2018**	12 months	DCB-PTA	105	FP	PAD (R2-4)	Dual 1 month; one antiplatelet drug daily
**Tepe 2008** *Thunder*	6 months	DCB-PTA	102	FP	PAD (R1-5)	Dual 1 month; ASS daily
**Tepe 2015** *IN.PACT-SFA*	12 months	DCB-PTA	331	FP	PAD (R2-4)	Dual 1 month (without stent) 3 months (bailout with stent); ASS >6month
**Tepe 2017** *CONSEQUENT*	24 months	DCB-PTA	153	FP	PAD (R2-4)	Dual >2months; ASS daily
**Werk 2008** *FemPac*	6 months	DCB-PTA	87	FP	PAD (R1-5)	Dual as longterm medication

**Werk 2012**<br>*PACIFIER*	12 months	DCB-PTA	85	FP	PAD (R2-5)	Dual >2 months
**Bosiers 2009**<br>*AMS Insight*	6 months	DES-PTA	117	IP	CLI (R4-5)	Dual 1 month;<br>ASS daily
**Bosiers 2012**<br>*DESTINY*	12 months	DCB-BMS	140	IP	CLI (R 4-5)	Dual 12 month
**Falkowski 2009**	6 months	DES-BMS	50	IP	CLI (R3-5)	Dual 6 month
**Haddad 2017**	12 months	DCB-PTA	93	IP	CLI (R>4)	Dual 3 month;<br>ASS daily
**Katsanos 2016**	12 months	DES-PTA	165	IP	PAD (R(2)3-5)	PTA-group: ASS<br>DES-group: Dual 6 month
**Liistro 2013**<br>*DEBATE-BTK*	12 months	DCB-PTA	132	IP	CLI (R>4) + DM	Dual 1 month;<br>ASS daily
**Mustapha 2019**<br>*Lutonix BTK trial*	6 months	DCB-PTA	442	IP	PAD (R3-5)	NA
**Rand 2011**<br>*InPERIA II Trial*	9 months	DES-PTA	88	IP	CLI (R4-5)	PTA-group: ASS;<br>DES-group: Dual 1 month
**Rastan 2012**	3 years	DES-BMS	161	IP	PAD (R3-5)	Dual 6 month
**Scheinert 2012**<br>*ACHILLES*	12 months	DES-PTA	200	IP	PAD (R3-5)	PTA-group: ASS<br>DES-group: Dual 6 month
**Siablis 2014**<br>*IDEAS*	6 months	DCB-DES	50	IP	PAD (R3-6) + Long Segment	Dual 6 month

**Spreen 2017**<br>*PADI*	5 years	DES-PTA	137	IP	CLI (R>4)	C 6 month;<br>Carbasalate Calcium daily
**Zeller 2015**<br>*BIOLUX P-II*	12 months	DCB-PTA	68	IP	PAD (Claudication or CLI)	Dual 1 month;<br>BMS-bailout: 3 month
**Zeller 2020**<br>*IN.PACT DEEP*	5 years	DCB-PTA	358	IP	CLI	NA

Table S2 Patient characteristics

Abbreviations: No. (number); R (Rutherfort); CLI (chritical limb ischaemia); NA (not available)

Author, year, acronym	Mean Age	No. (%) female sex	DM	Current Smoking	Hyperlipidaemia	Hypertension	Claudication (R2-3)	CLI (R4-6)
**Bausback 2017** *RANGER-SFA*	67,5	28,5	40	46	70	84	102	NA
**Bausback 2019** Real PTX	68,9	32	48	61	107	120	130	20
**Dake 2011** *Zilver PTX*	67,8	35,2	216	NA	246	404	430	41
**Duda 2006** *SIROCCO*	66,1	28	36	36	59	64	NA	NA
**Falkowski A. 2020**	65,8	36,5	98	NA	160	208	NA	NA
**Iida 2018** *MDT-2113 SFA*	73,8	23	58	28	NA	NA	96	NA
**Jia 2016** *AcoArt-I*	65,8	27	111	62	56	134	116	84
**Krisnan 2017** *ILLUMENATE Pivotal*	69	40	151	NA	266	281	287	13
**Liistro 2013** *DEBATE-SFA*	75	30,9	77	53	60	92	39	55
**Rosenfield 2015** *LEVANT-2*	68,4	36	204	165	421	422	438	38
**Scheinert 2014** *LEVANT-1*	68,5	37	48	35	65	92	94	7

**Scheinert 2015** *BIOLUXP-1*	70,8	43,3	20	41	37	44	29	6
**Schroeder 2017** *ILLUMENATE EU RCT*	68	30	109	158	186	233	287	5
**Steiner 2018**	67,5	28,5	40	46	70	84	NA	NA
**Tepe 2008** *Thunder*	68,3	34,3	76	37	100	128	NA	NA
**Tepe 2015** *IN.PACT-SFA*	67,8	33,7	143	125	277	299	313	18
**Tepe 2017** *CONSEQUENT*	68,1	32	56	73	83	120	NA	NA
**Werk 2008** *FemPac*	68,8	40	41	36	50	69	79	5
**Werk 2012** *PACIFIER*	71	38,5	32	NA	44	60	87	4
**Bosiers 2009** *AMS Insight*	73,9	45	82	50	77	102	0	117
**Bosiers 2012** *DESTINY*	75,5	51	77	18	53	96	0	140
**Falkowski 2009**	69,4	21	20	22	18	31	34	16
**Haddad 2017**	-	-	89	68	85	79	0	93
**Katsanos 2016** *Achilles*	73,4	57	129	65	146	181	NA	NA
**Liistro 2013** *DEBATE-BTK*	74,6	26	132	20	39	98	0	132
**Mustapha 2019**	72,9	136	310	62	341	412	42	400

**Rand 2011**<br>*InPERIA II Trial*	71,8	30	69	NA	NA	NA	0	88
**Rastan 2012**	72,9	54	87	46	123	145	86	75
**Scheinert 2012**<br>*ACHILLES*	73,4	57	129	65	146	181	NA	NA
**Siabilis 2014**<br>*IDEAS*	71,5	12	35	15	23	25	NA	NA
**Spreen 2017**<br>*PADI*	73,6	41	87	33	NA	NA	0	137
**Zeller 2015**<br>*BIOLUX P-II*	71,3	15	48	10	49	62	16	52
**Zeller 2020**<br>*IN.PACT DEEP*	73,3	92	263	NA	255	320	1	357

Figure S1 Results of the network analysis of mortality endpoints in full model analysis and subgroups

The forest plot depicts risk ratio and 95% confidence interval with the different endovascular treatment strategies compared to PTA. Abbreviations: MAE (Major Adverse Events); BMS (Bare Metal Stent), DES (Drug Eluting Stent), DCB (Drug Coated Balloon), PTA (Percutan Transluminal Angioplasty), RR (Risk Ratio), CI (Confidence Interval)

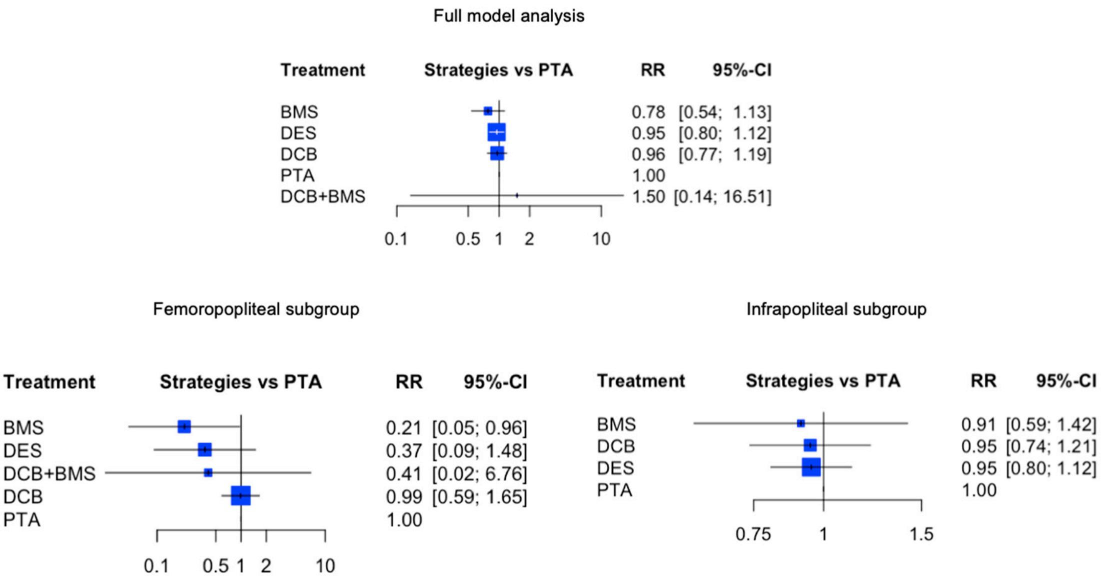

Figure S2: Net Heat Plot Analysis of Treatment Efficacy in Peripheral Arterial Disease

The figure depicts the network meta-analysis heat plots of the major advers event analyses. These plots were generated using a random-effects model with the netheat function from the netmeta package in R, which visually represents the direct and indirect comparisons across different interventions. Each square's intensity corresponds to the amount of information contributing to the network estimates for that comparison. Abbreviations: RR (Relative Risk), CI (Confidence Interval), BMS (Bare Metal Stent), DCB (Drug-Coated Balloon), DCB+BMS (Drug-Coated Balloon plus Bare Metal Stent), DES (Drug-Eluting Stent), PTA (Percutaneous Transluminal Angioplasty).

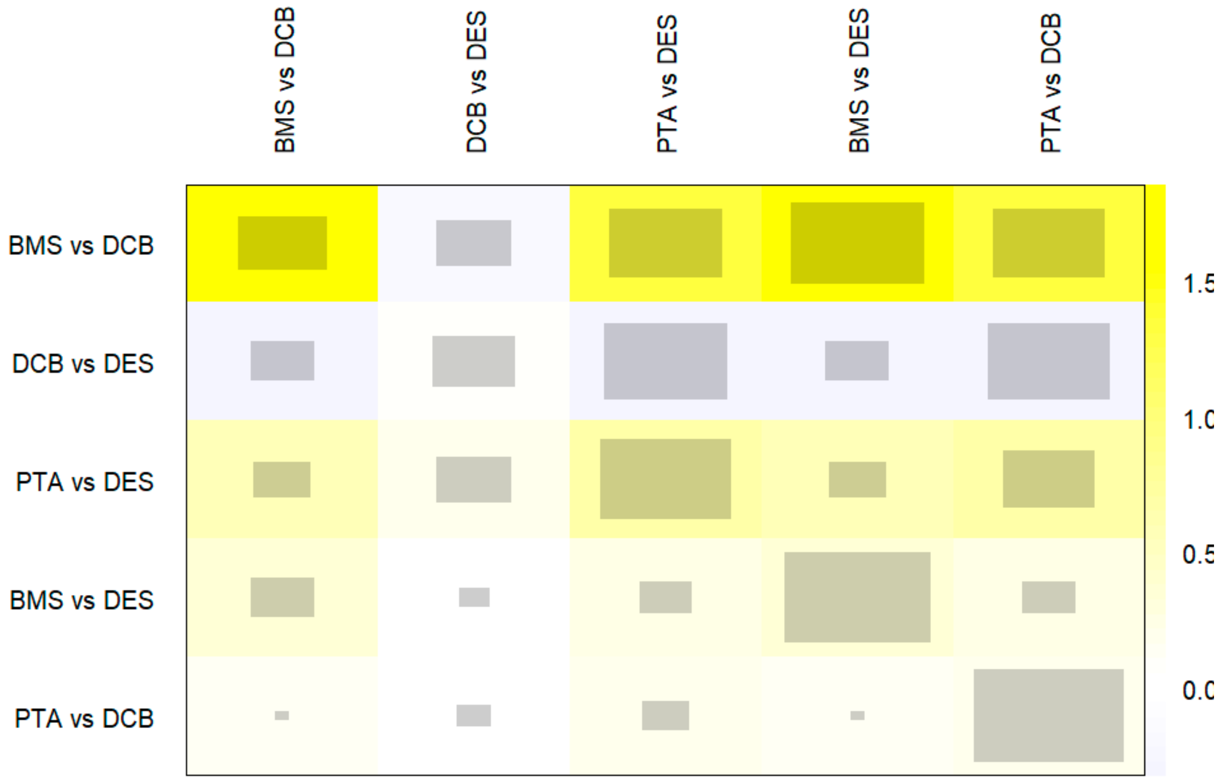

Figure S3: Net Split Plot Analysis of Major Adverse Events in Full Model Analysis

This figure presents the net split plot analysis for the major adverse event (MAE) endpoint based on a full model analysis of peripheral arterial disease interventions. The net split plot visualizes the contribution of direct and indirect evidence to the overall network estimates for MAE.

The plot is composed of paired comparisons between interventions, with the size of the squares indicating the weight of the direct evidence and the connecting lines representing the indirect evidence. A larger square size denotes a greater influence of the direct evidence on the network meta-analysis result for that comparison.

Abbreviations: RR (Relative Risk), CI (Confidence Interval), BMS (Bare Metal Stent), DCB (Drug-Coated Balloon), DCB+BMS (Drug-Coated Balloon plus Bare Metal Stent), DES (Drug-Eluting Stent), and PTA (Percutaneous Transluminal Angioplasty).

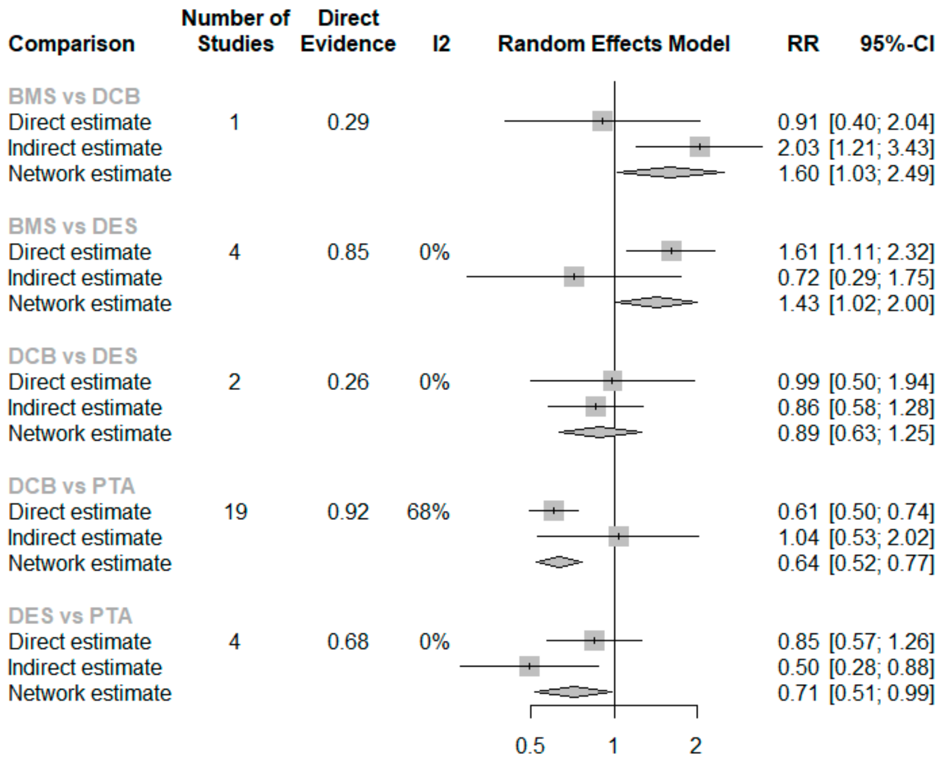

Figure S4: Comparison Adjusted Funnel Plot for Major Adverse Event Endpoint in Full Model Analysis

The figure presents a comparison adjusted funnel plot generated from the full model analysis of the major adverse event (MAE) endpoint, as part of a network meta-analysis evaluating the comparative effectiveness of different interventions for peripheral arterial disease (PAD). The plot is designed to detect publication bias and small-study effects within the network meta-analysis. Each point represents a study comparison, colored according to the treatment type. The vertical line indicates no effect, and the diagonal lines represent the expected distribution of studies in the absence of heterogeneity or publication bias. The Begg and Mazumdar rank correlation test uses the correlation between the ranks of effect sizes and the ranks of their variances is applied to assess the symmetry of the funnel plot, which helps to determine the presence of publication bias

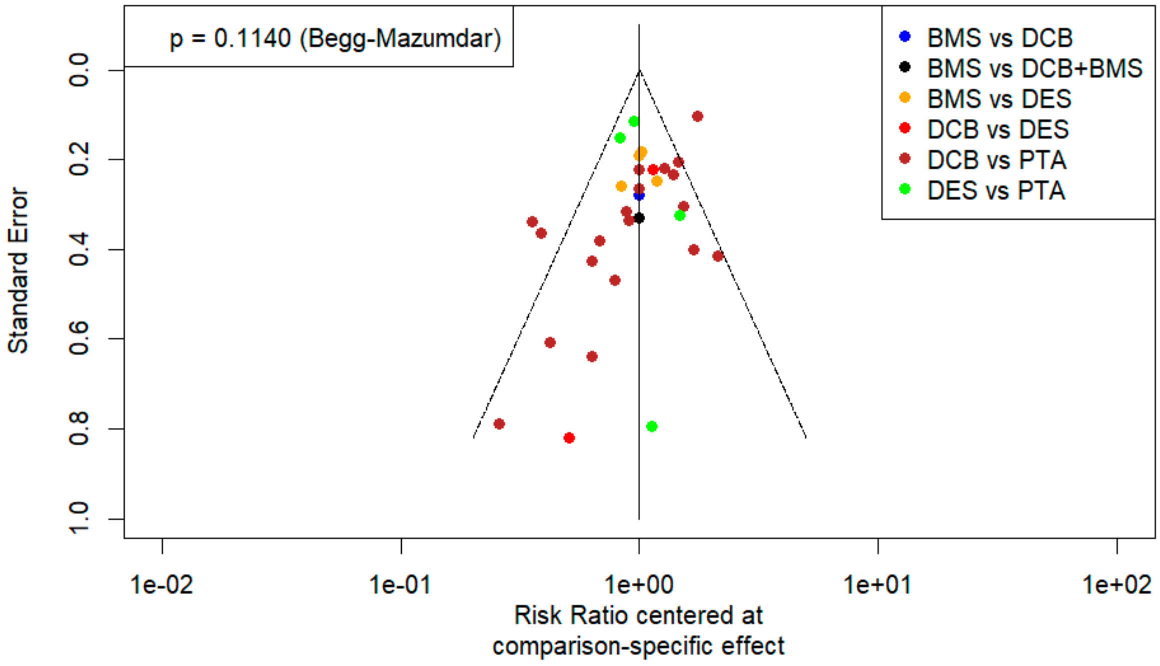

Figure S5: Risk of bias in studies

Risk of bias summary. Green circles with a "+" sign indicate low risk; red circles with a "−" sign indicate high risk; and yellow circles with a "?" indicate unclear risk.

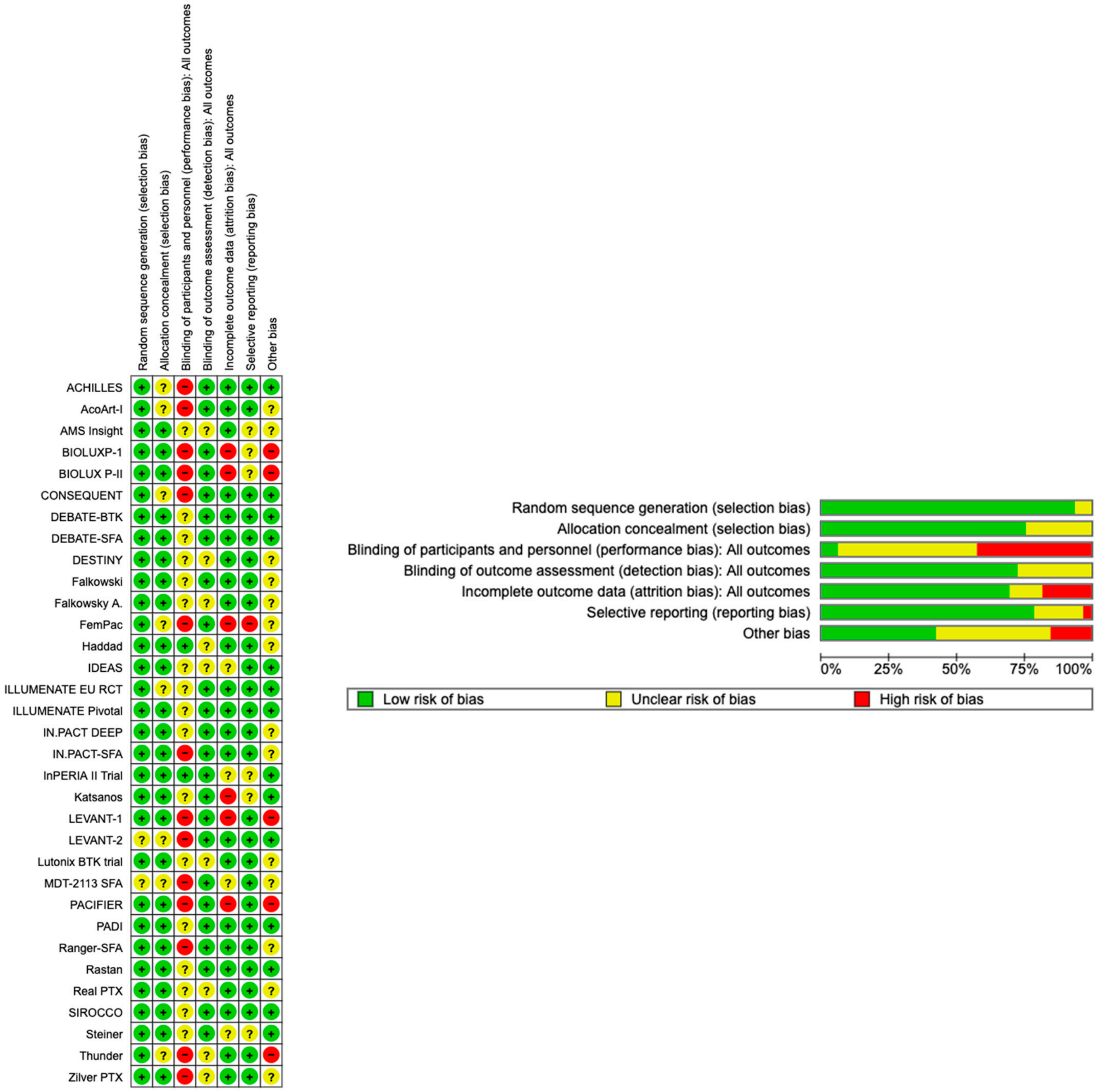

Supplement: Supplementary file 1 [file jcm-13-01024-s001.zip › jcm-2811628-supplementary.pdf]
